# New Officinal Preparations

**Published:** 1851-08

**Authors:** 


*New Officinal Preparations.*—We have examined the following new officinal remedies prepared by A. I. Mathews, apothecary, after formulæ contained in the late edition of the United States Pharmacopœia:

*Liquor Magnesiæ Citratis.* This is an excellent laxative preparation, agreeable to the taste, peculiarly adapted to children, and patients who have an invincible repugnance to nauseous doses.

*Syrupus Acaciæ.* A good vehicle for remedies.

*Syrupus Krameriæ.* A very pleasant astringent.

*Syrupus Sennæ.*

*Syrupus Pruni Virginianæ.*

The above articles will be found very useful additions to the list of officinal preparations.

